# Spectral characteristics of urine from patients with end-stage kidney disease analyzed using Raman Chemometric Urinalysis (Rametrix)

**DOI:** 10.1371/journal.pone.0227281

**Published:** 2020-01-10

**Authors:** Ryan S. Senger, Meaghan Sullivan, Austin Gouldin, Stephanie Lundgren, Kristen Merrifield, Caitlin Steen, Emily Baker, Tommy Vu, Ben Agnor, Gabrielle Martinez, Hana Coogan, William Carswell, Varun Kavuru, Lampros Karageorge, Devasmita Dev, Pang Du, Allan Sklar, James Pirkle, Susan Guelich, Giuseppe Orlando, John L. Robertson

**Affiliations:** 1 Department of Biological Systems Engineering, Virginia Tech, Blacksburg, Virginia, United States of America; 2 Department of Chemical Engineering, Virginia Tech, Blacksburg, Virginia, United States of America; 3 DialySenors, Inc., Blacksburg, Virginia, United States of America; 4 Veteran Affairs Medical Center, Salem, Virginia, United States of America; 5 Department of Statistics, Virginia Tech, Blacksburg, Virginia, United States of America; 6 Lewis-Gale Medical Center, Salem, Virginia, United States of America; 7 Department of Internal Medicine–Nephrology, Wake Forest University Baptist Medical Center, Winston-Salem, North Carolina, United States of America; 8 Valley Nephrology Associates, Roanoke, Virginia, United States of America; 9 Department of Surgical Sciences–Transplant, Wake Forest University Baptist Medical Center, Winston-Salem, North Carolina, United States of America; 10 Department of Biomedical Engineering and Mechanics, Virginia Tech, Blacksburg, Virginia, United States of America; 11 Virginia Tech-Carilion School of Medicine and Research Institute, Blacksburg, Virginia, United States of America; Center for Molecular Biotechnology, ITALY

## Abstract

Raman Chemometric Urinalysis (Rametrix^TM^) was used to discern differences in Raman spectra from (i) 362 urine specimens from patients receiving peritoneal dialysis (PD) therapy for end-stage kidney disease (ESKD), (ii) 395 spent dialysate specimens from those PD therapies, and (iii) 235 urine specimens from healthy human volunteers. Rametrix^TM^ analysis includes spectral processing (e.g., truncation, baselining, and vector normalization); principal component analysis (PCA); statistical analyses (ANOVA and pairwise comparisons); discriminant analysis of principal components (DAPC); and testing DAPC models using a leave-one-out build/test validation procedure. Results showed distinct and statistically significant differences between the three types of specimens mentioned above. Further, when introducing “unknown” specimens, Rametrix^TM^ was able to identify the type of specimen (as PD patient urine or spent dialysate) with better than 98% accuracy, sensitivity, and specificity. Rametrix^TM^ was able to identify “unknown” urine specimens as from PD patients or healthy human volunteers with better than 96% accuracy (with better than 97% sensitivity and 94% specificity). This demonstrates that an entire Raman spectrum of a urine or spent dialysate specimen can be used to determine its identity or the presence of ESKD by the donor.

## Introduction

The chemical composition, physical characteristics, and types/amounts of suspended materials in urine change when kidney (and systemic) disease is present [1–4]. In this study, Raman Chemometric Urinalysis (Rametrix^TM^) [5–8] was used to determine if differences in molecular spectra could be detected in the following specimen types: (i) urine from healthy human volunteers, (ii) urine from patients undergoing peritoneal dialysis (PD) therapy for end-stage kidney disease (ESKD) and (iii) spent dialysate from these patients receiving PD therapy. Rametrix^TM^ relies on Raman spectroscopy and a biological region of the spectrum (400–1,800 cm^-1^) that is composed of spectral signatures of the thousands of molecules known to the urine metabolome [9,10]. Furthermore, Rametrix^TM^ uses off-the-shelf Raman spectrometers, which are becoming more affordable, low-profile, and conducive to clinical laboratory use [11].

In the past, sophisticated analyses, including mass spectrometry, liquid/gas chromatography, and kinetic nephelometry have been used to detect analytes (i.e., “biomarkers”) in urine associated with metabolism or disease [3,12–15]. In addition, urine contains byproducts of therapeutics that help clinicians monitor and adjust therapies [16,17] and can indicate environmental and occupational toxin exposure [18].

Urine biomarker studies to detect chronic kidney disease have been conducted almost exclusively in research settings. For example, Zürbig and co-workers [19] used capillary electrophoresis, coupled online to electrospray ionization time-of-flight mass spectrometry (CE-MS), to identify polypeptide patterns in human urine. However, proteomic spectral patterns in urine, as biomarkers for kidney disease, have not been accepted widely as diagnostic tools, and proteomic patterns identified in that study could not be translated to specific molecules with physiological and pathophysiological significance. This significantly dampened enthusiasm for proteomic spectral pattern recognition as a tool to diagnose and study genitourinary tract disease.

For these and other reasons, biomarker and “-omics” technologies are used rarely (or are not readily available) by caregivers in patient care settings. This is due to expense, the daunting requirement for advanced technology (such as mass spectrometry), expertise required for interpretation of results, and a lack of assay validation with large datasets of normal and abnormal specimens. In fact, the complexity of both acute and chronic kidney diseases, and other genitourinary tract pathologies, makes large dataset sampling and validation both unlikely and cost-prohibitive [20–23]. Rametrix^TM^, on the other hand, provides robust spectral information about the urine metabolome, using instrumentation that is inexpensive. To achieve useful results, minimal technician training is required, and Rametrix^TM^ software automates analysis. This has enabled studies, such as this and others [5–8,24–27], using very large datasets, to discover spectral differences between normal and abnormal (e.g., diseased, stressed, decayed, chemically treated, etc.) samples.

Previously, we reported the Rametrix^™^ analysis of 235 urine specimens obtained from consented, healthy, human volunteers [8]. This work identified common Raman spectral characteristics seen in urine specimens obtained from healthy persons of both sexes and between 18–70 years of age. We focused on determining the variation (or lack thereof) in spectral signatures from several subsets of individuals over 30 days, comparing data from single time-point (first voided sample in the morning) collections. As part of this work, we noted significant effects of sex and age of the donor but negligible effects of menstruation on Raman spectral characteristics.

Here, we describe the results of Rametrix^™^ analysis of 362 urine specimens collected from patients with ESKD, who had residual renal function and were undergoing PD treatments. We also describe Rametrix^TM^ analysis of 395 specimens of spent dialysate from those patients undergoing PD treatments. These were then compared with the 235 urine specimens from healthy human volunteers [8] using Rametrix^TM^. All collections and data analysis were devoted to testing the hypothesis that “Rametrix^™^ analysis of urine specimens can discern significant molecular composition differences between (i) PD patient urine and spent dialysate and (ii) PD patient urine and urine from healthy individuals.” The study to discern the molecular differences between PD patient urine and spent dialysate was designed to demonstrate the capabilities of Rametrix^TM^ analysis and provide a basis for future studies that look at patient-specific molecules that are removed by PD or the kidneys. The study to discern PD patient urine from urine of healthy individuals was designed to identify new patients who should receive PD therapy and to be able to monitor the progress of those patients receiving PD therapy.

## Materials and methods

### Informed consent

Informed written consent for the collection of urine specimens from healthy human volunteers was obtained under research protocol VT15-703, approved and administered by the Virginia Tech Institutional Review Board. Informed written consent for the collection of urine and dialysate specimens from patients undergoing dialysis therapies was obtained under research protocol RPP/177151.2, approved and administered by Frenova (Fresenius Renal Research; 920 Winter Street, Waltham, MA 02451). In accordance with these protocols, specimens were de-identified and assigned a code at the time of collection.

### Description of study population and sampling

Three groups of specimens were compared in this study: (i) urine from patients undergoing PD therapy for ESKD, (ii) the spent dialysate from those PD therapies, and (iii) urine from healthy human volunteers. A full analysis of the healthy human volunteer urine dataset has been published [8]. Briefly, 235 urine specimens were collected from 48 (39 females, 9 males) healthy human volunteers with no history or evidence of renal disease. Volunteers were also free of infectious or degenerative disease at the time of sample collection. The age range of the healthy volunteer population was 18–70 years; 87.5% of volunteers were of ages 19–22 years, and the median age was 21 years.

For patients undergoing PD therapies, 362 urine specimens were collected from 96 patients, and 395 spent dialysate specimens from 115 patients comprised the dataset. Patients had advanced ESKD and were undergoing PD treatment. Patients ranged in age from 24–90 years old. The mean age was 60 years, and the median age was 63.5 years. Multiple collections (4–8 separate collections) were available from multiple patients, allowing repetitive measurements and correlations over a protracted course of PD therapy (18 months).

### Specimen collection and storage

Specimens were collected at the time of routine PD adequacy evaluation (generally every 1–3 months) over a period ranging from 18–24 months. For routine adequacy testing, patients collected all urine produced in a 24-hour period and also collected all spent dialysate from multiple cycles of treatment that occurred over 24 hours. These urine samples and spent dialysate collections were brought to the dialysis center, where aliquots of urine and spent dialysate were transferred into sterile specimen cups and then immediately frozen to -15°C. Both urine and spent dialysate specimens were stored at this temperature until analyzed. Urine specimens from healthy human volunteers were stored immediately at -35°C until analyzed.

We previously determined the suitability of collection and storage conditions in a separate study of urine stability [7] and adhered to the guidelines set forth in that study. Unused portions of urine and spent dialysate specimens were stored at -35°C for the duration of the study and re-analyzed, as needed.

### Analytical standards

Surine^™^ Urine Negative Control (Dyna-Tek Industries, Lenexa, KS) was used as a control standard for urinalysis. Unused dialysate (obtained from Valley Nephrology Associates; Roanoke, VA) was also used as a reference control in this study.

### Raman methodology and measurements

Previously published experimental methods were used [6, 7]. Briefly, an Agiltron PeakSeeker^™^ dispersive Raman spectrometer (Woburn, MA) was used, and all specimens were Raman scanned as bulk liquid samples in 1.5 mL glass vials at 25°C using 785 nm (30 mW) laser excitation for 30 s with spectral resolution of 8 cm^-1^. A minimum of 10 scans were collected per specimen and averaged.

### Computational methodology

Previously published computational methods were also used [7,8]. Spectral processing and analyses were performed with the Rametrix^TM^ LITE [5], Rametrix^TM^ PRO [6], and Statistics and Machine Learning Toolboxes were used with MATLAB r2018A (The MathWorks, Inc.; Natick, MA). Raman spectra were truncated to 400–1,800 cm^-1^, baseline corrected using the Goldindec algorithm [28], and vector normalized. Principal component analysis (PCA) and discriminant analysis of principal components (DAPC) models were constructed using the Rametrix^TM^ LITE Toolbox, and DAPC models were tested by leave-one-out analysis with the Rametrix^TM^ PRO Toolbox. 1-Way ANOVA and pairwise comparisons using Tukey’s honestly significant difference (HSD) procedure were performed in MATLAB.

### Public availability

The Rametrix^TM^ LITE Toolbox is available through GitHub under an MIT licensing agreement (https://github.com/SengerLab/RametrixLITEToolbox). The Rametrix^TM^ PRO Toolbox is also available through GitHub under similar licensing agreement (https://github.com/SengerLab/RametrixPROToolbox). Raman scans of urine from ESKD patients are available with these tools. Data used for statistical analyses are also available through GitHub (https://github.com/SengerLab/Raman-Scans/tree/ESKD).

## Results

### Relevant questions

Raman spectroscopy and Rametrix^TM^ analysis was used to answer the following questions:

1. Are PD patient urine and spent dialysate different?
2. Are PD patient urine and urine from healthy volunteers different?
3. Can Rametrix^TM^ identify “unknown” specimens correctly?

### Raman spectroscopy of PD patient urine and spent dialysate

**Fig 1:**
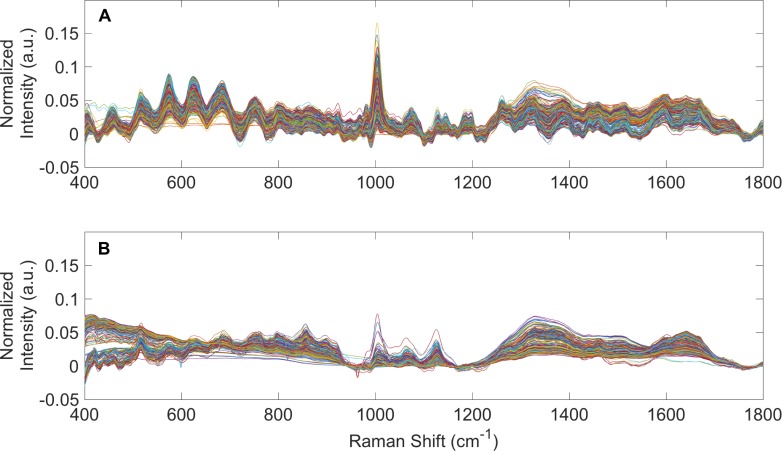
Raman spectra of PD patient urine and spent dialysate. (A) Averaged, baselined, and vector normalized Raman spectra from 362 urine specimens obtained from patients receiving PD therapy for ESKD. (B) Averaged, baselined, and vector normalized Raman spectra from 395 spent PD dialysate specimens.

Raman spectra from 362 urine specimens and 395 spent dialysate specimens from PD patients were averaged (per specimen), baseline corrected using the Goldindec algorithm [27], and vector normalized. These were plotted together in Fig 1A (PD patient urine) and Fig 1B (spent dialysate). Raman spectra of the 235 urine specimens from healthy human volunteers were processed similarly and published elsewhere [8]. Apparent in Fig 1, there are observable differences between Raman spectra of PD patient urine and spent dialysate. In particular, there are clear differences in the urea (1,002 cm^-1^) [10,29] content of these specimens. While all specimens seemed to show a basic spectral signature of urine or spent dialysate, there were considerable differences between the individual spectra within these specimen types. These were explored further by PCA and statistical tests to provide quantitative metrics.

### Principal component analysis

**Fig 2:**
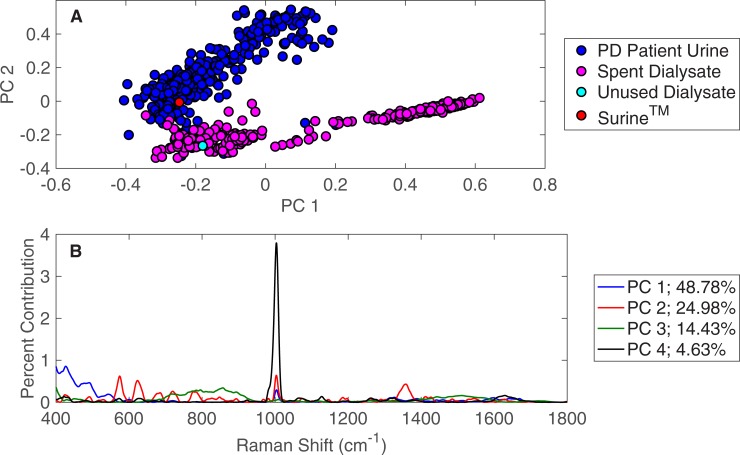
PCA of PD patient urine and spent dialysate. (A) PCA results for Raman spectra of 362 urine specimens obtained from patients receiving PD therapy for ESKD and 395 spent dialysate specimens. (B) Contributions of Raman shifts leading to separations among principal components.

The PD patient urine specimens were compared against the spent dialysate specimens using PCA in the Rametrix^TM^ LITE Toolbox. The first two principal components are plotted in Fig 2A. Here, significant separation is observed between the two specimen types (i.e., PD urine and spent dialysate). PCA was applied with two controls: (i) Surine^TM^ as a urinalysis standard and (ii) unused dialysate. Surine^TM^ clustered with the urine specimens, and the unused dialysate clustered with the spent dialysate, showing some similarity between these specimen types. The Rametrix^TM^ LITE Toolbox also identifies Raman shifts that lead to the separation of clusters in PCA [5]. These can be traced back to individual molecules by scanning individual standards, metabolomic knowledge [9], and spectral libraries [10]. For the PD patient urine and spent dialysate dataset, these Raman shift contributions are shown in Fig 2B. Contributions from the top four principal components are shown, and together, these represent over 92% of the dataset variance. Again, the Raman shift at 1,002 cm^-1^ was the most dominant, present in all principal components, and is representative of urea in urine and dialysate specimens. Other notable Raman shifts in Fig 2B include creatinine (680 cm^-1^) [30,31] and glucose (1,071 cm^-1^; 1,117 cm^-1^; others) [8,32]. Research is ongoing to validate more Raman shifts in this and similar plots using Raman scans of standards and metabolomic analysis. We note, however, the chemometric approach of Rametrix^TM^ (described in the following sections) allow meaningful results to be obtained without the assignment of analytes to individual Raman bands.

**Fig 3:**
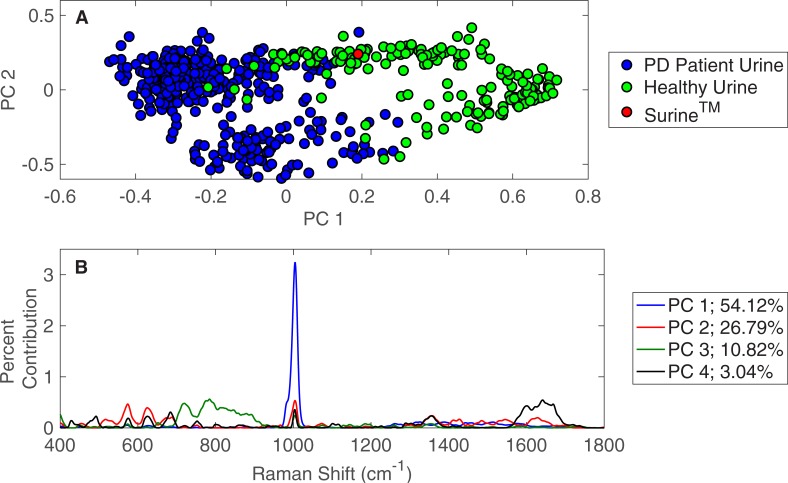
PCA of PD patient urine and urine from healthy individuals. (A) PCA results for Raman spectra of 362 urine specimens obtained from patients receiving PD therapy for ESKD and 235 urine specimens from healthy individuals. (B) Contributions of Raman shifts leading to separations among principal components.

The PD patient urine specimens were also compared against the urine specimens from healthy human volunteers by PCA. These results are shown in Fig 3A and show the separation of clusters between the two specimen types, suggesting significant spectral differences and molecular compositions. This time, the Surine^TM^ urinalysis control clustered with urine specimens from healthy volunteers, rather than those from PD patients. The spectral differences (i.e., signal intensities at each Raman shift) that lead to the observed separation between urine specimens from PD patients and healthy volunteers in PCA are given in Fig 3B. The top four principal components represent more than 94% of the dataset variance in this case. Here, urea (1,002 cm^-1^) is dominant in the first principal component (PC 1). Again, creatinine and glucose are apparent in Fig 3B, and additional Raman shift contributions are present relative to the comparison of PD patient urine and spent dialysate (Fig 2B).

### Statistical analyses

The entire dataset (consisting of Raman scans of healthy human volunteer urine, PD patients urine, and spent dialysate) was analyzed by 1-way ANOVA to determine if the type of specimen was statistically significant. To do this, the spectra were each quantified to a single numerical value through calculation of the total principal component distance (TPD). This calculation has been explained and demonstrated previously [7,8]. Briefly, TPD represents how closely the Raman spectrum of a specimen resembles that of Surine^TM^. To calculate this, the distance formula is applied between the top four principal components of a specimen and those of Surine^TM^. This procedure reduces a data-rich Raman spectrum down to a single numerical value, which allows statistical tests to be applied. In particular, a 1-way ANOVA test of TPD values for all specimen types (i.e., healthy human urine, PD patient urine, or spent dialysate) returned a p-value less than 0.001, which confirmed that the type of specimen was statistically significant. Results of pairwise comparison tests with Tukey’s HSD procedure are shown in Table 1. Statistical significance (p < 0.001) was obtained when comparing (i) PD patient urine with spent dialysate, (ii) PD patient urine with healthy human volunteer urine, and (iii) PD patient spent dialysate with healthy human volunteer urine. Results confirm these types of specimens are all different from one another. Other pairwise comparisons did not return statistical significance likely because only single controls (Surine^TM^ and unused dialysate) were included in the dataset.

**Table 1:** Pairwise comparisons using Tukey’s HSD procedure.

Specimen 1	Specimen 2	p-Value
Spent PD Dialysate	PD Patient Urine	< 0.001
Spent PD Dialysate	Unused Dialysate	0.906
Spent PD Dialysate	Healthy Urine	< 0.001
Spent PD Dialysate	Surine^TM^	0.0517
PD Patient Urine	Unused Dialysate	1.00
PD Patient Urine	Healthy Urine	< 0.001
PD Patient Urine	Surine^TM^	0.346
Unused Dialysate	Healthy Urine	0.905
Unused Dialysate	Surine^TM^	0.689
Healthy Urine	Surine^TM^	0.874

### Discriminant analysis of principal components models

**Fig 4:**
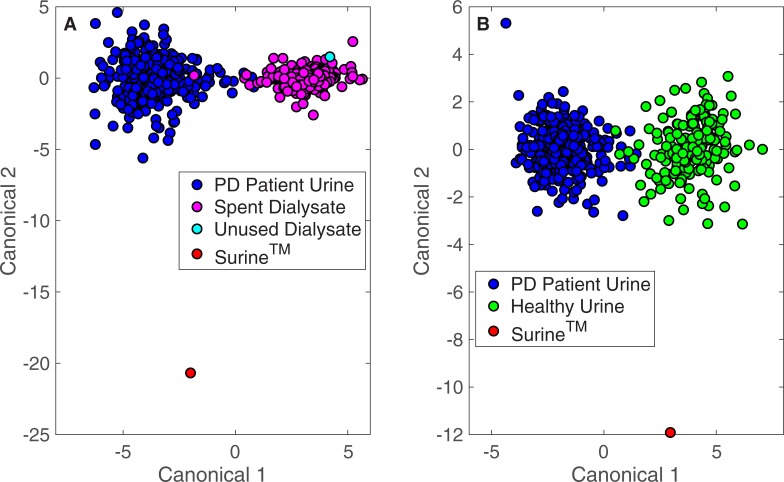
DAPC of PD patient urine, spent dialysate, and urine from healthy individuals. DAPC results for models made with 50 principal components. (A) 362 urine specimens obtained from patients receiving PD therapy for ESKD and 395 spent dialysate specimens. (B) 362 urine specimens obtained from patients receiving PD therapy for ESKD and 235 urine specimens from healthy individuals.

The Rametrix^TM^ LITE Toolbox was used to generate DAPC models for datasets consisting of Raman scans of: (i) PD patient urine and spent dialysate and (ii) PD patient urine and urine from healthy human volunteers. Surine^TM^ was included as a control in both models, and unused dialysate was used as an additional control for the model containing PD patient spent dialysate. The Rametrix^TM^ PRO Toolbox was then used to evaluate the predictive capabilities of these models using a leave-one-out build/test validation routine. DAPC model clustering is shown in Fig 4 for both models when 50 principal components were used in model construction. Good separation of clusters (with some overlap) was observed in both cases. Surine^TM^ was separated from all clusters in both models, and the unused dialysate standard clustered with the PD patient spent dialysate specimens.

The prediction capabilities (from “leave-one-out” build/test routines) of the DAPC models are shown in Tables 2 and 3. The DAPC models were built with different numbers of principal components to ensure enough dataset variance was included in the models and to test for model overfitting. DAPC models were evaluated in terms of prediction accuracy (the percentage of data points predicted correctly), sensitivity (the true positive percentage), and specificity (the true negative percentage). Results in Table 2 convey Rametrix^TM^ can determine the identity of an “unknown” specimen from a PD patient as being either urine or spent dialysate with very high confidence. Greater than 98% accuracy, sensitivity, and specificity were obtained for a DAPC model consisting of 10 principal components. For all DAPC models tested, the accuracy, sensitivity, and specificity values exceeded 97%. This high level of confidence in identifying the type of sample (i.e., urine or spent dialysate) is unsurprising given the clear differences in Raman spectra shown in Fig 1. For determining whether a urine specimen originated from a PD patient or healthy human volunteer, Rametrix^TM^ prediction results are given in Table 3. Better than 96% prediction accuracy (with better than 97% sensitivity and 94% specificity) was obtained for the DAPC model constructed with 11 principal components. Using 50 principal components led to increased accuracy and sensitivity with decreased specificity, which is characteristic of model over-fitting.

**Table 2:** Rametrix^TM^ PRO results showing the ability to predict whether an unknown specimen from a PD patient is urine or spent dialysate.

Percent Variability Explained by Principal Components	Number of Principal Components used in DAPC Model	Accuracy*	Sensitivity*	Specificity*
90%	4	97.8%	98.6%	97.0%
95%	5	97.2%	98.1%	96.5%
99%	10	98.7%	98.9%	98.5%
99.9%	50	98.2%	98.9%	97.5%

*Predictions were from a leave-one-out training/testing routine.

**Table 3:** Rametrix^TM^ PRO results showing the ability to predict whether an unknown urine specimen came from a PD patient or healthy human volunteer.

Percent Variability Explained by Principal Components	Number of Principal Components used in DAPC Model	Accuracy*	Sensitivity*	Specificity*
90%	3	94.0%	95.6%	90.8%
95%	5	93.1%	92.8%	93.6%
99%	11	96.1%	97.0%	94.2%
99.9%	50	97.4%	99.7%	92.5%

*Predictions were from a leave-one-out training/testing routine.

## Discussion

Rametrix^TM^ has demonstrated the ability to discern effectively among (i) urine from PD patients (ESKD), (ii) spent dialysate from their PD therapies, and (iii) urine from healthy human volunteers. Cluster separations (according to specimen type) were readily apparent in PCA and DAPC model plots, and the conversion of spectral data to TPD values for statistical analyses also confirmed these differences were statistically significant. We have begun the process of identifying molecules responsible for these differences, and we hypothesize this may result in a new set of biomarkers for ESKD and earlier stages of chronic kidney disease. However, we were able to show that the entire Raman spectrum of a specimen can be used (i.e., chemometrics) to determine its type (i.e., urine or dialysate) or the state of the donor (i.e., healthy human or PD patient). The leave-one-out build/test validations of Tables 2 and 3 are particularly important because they describe how well Rametrix^TM^ can perform with “unknown” specimens. Of course, the long-term vision with Rametrix^TM^ is not to be able to discern whether an unknown specimen is urine or dialysate but to be able to screen for the presence of incipient disease and patient-specific PD responses. For example, Rametrix^TM^ could be used to determine whether PD therapies are patient-specific and if there are variations among successive treatments. If so, how do these affect long-term patient outcomes? Comparing urine from PD patients to that of healthy human volunteers is a first crucial step in this process. The PD patients used in this study have ESKD; thus, the differences between urine from these patients and healthy human volunteers should be significant and easily detectable by Rametrix^TM^, as was confirmed by this study. The next steps in Rametrix^TM^ development are to be able to detect earlier stages (i.e., G1-4) and track patient progress over longer periods of time.
